# Revisiting hydrolytic mechanisms of bacterial RNase HI: Towards clarification of single mobile Mg^2+^ action in nuclease reactions

**DOI:** 10.2142/biophysico.bppb-v23.0014

**Published:** 2026-04-18

**Authors:** Kosuke Morikawa, Takuji Oyama, Masayuki Oda

**Affiliations:** 1 Graduate School of Life and Environmental Sciences, Kyoto Prefectural University, Kyoto 606-8522, Japan; 2 Faculty of Life and Environmental Sciences, University of Yamanashi, Kofu, Yamanashi 400-8510, Japan

**Keywords:** crystal structure, enzyme, Mg^2+^-dependent hydrolytic mechanism

## Abstract

Cellular nuclei contain various nucleases that play major roles in gene expression and regulation. They are classified into various categories based on their three-dimensional structures and specificities. Since the discovery of ribozyme in the early 1980, one nuclease class has attracted significant attention. The cleavage reactions of these nucleases essentially require Mg^2+^ cations and produce a 5'-phosphate nucleotide and a 3'-nucleoside at the cleavage site of polynucleotides, similarly to the reaction of ribozyme. In particular, Drs. Thomas A. Steitz and Joan A. Steitz proposed an attractive and general model for the phosphoryl transfer reaction. This was later named the “two-metal-binding mechanism”. In accordance with recent developments in cryo-electron microscopy (cryo-EM), many structural reports published about Mg^2+^-dependent nucleases or spliceosomes support the “two-metal-binding mechanism” for hydrolytic schemes. However, the direct visualization of Mg^2+^ on electron-density maps requires high resolution analyses, regardless of X-ray crystallography or cryo-EM. Furthermore, recent biochemical studies combined with structural data support the similarity in hydrolytic mechanisms between ribonuclease HI (RNase HI) and restriction endonucleases. Since the late 2000s, molecular dynamics approaches have also appeared to support the hydrolytic mechanism of single mobile metals. This review aims to critically reconsider Mg^2+^-dependent hydrolytic mechanisms, with a particular focus on bacterial RNase HI.

## Significance

The issue of essential metal ion binding stoichiometry to ribonuclease HI, that is, one- or two-metal binding, remains controversial. In addition to our mutational and structural analyses, recent simulation studies have supported the notion that a single mobile metal for nucleases is critical to the hydrolytic mechanism. We reconsider the Mg^2+^-dependent hydrolytic mechanisms of nucleases, including ribonuclease HI, and discuss the limitations of conventional three-dimensional structural analyses, such as X-ray crystallography and cryo-electron microcopy. To understand the real view of enzyme function, direct observation of conformational fluctuations during catalytic processes would be required.

## Introduction

From the present perspective of molecular biology, biological individuals depend on the activities of various proteins including enzymes and nucleic acid molecules such as DNA and RNA. In the early 1980s, some RNA molecules, like enzymes, were discovered to exhibit catalytic activity, although they were previously believed to function only in information transfer. Although the concept of “ribozyme” was thus established, the major roles of ribozyme involve the cleavage of phosphodiester bonds dependent on Mg^2+^ cations. On the other hand, since the early days of molecular biology, many protein nucleases with similar catalytic reactions have been reported in prokaryotic and eukaryotic cells, and they have been clearly classified into various categories according to their three-dimensional (3D) structures and specificities. Among such categories, one nuclease class, in which cleavage reactions essentially require Mg^2+^ cations and produce a 5'-phosphate nucleotide and 3'-nucleoside at the cleavage site of polynucleotides, is particularly important, because, in both prokaryotes and eukaryotes, they are involved in gene expression and regulation, such as DNA replication, transcription, and DNA repair. Therefore, studies of their specific roles and catalytic mechanisms are central to cell biology. Notably, these Mg^2+^-requiring nucleases and ribozymes appear to undergo catalytic reactions via similar phosphoryl transfer reactions. In fact, Drs. Thomas A. Steitz and Joan A. Steitz proposed an attractive hypothetical model, in which most polynucleotides, such as RNA and DNA in nuclei, are hydrolyzed through a general mechanism, where the simultaneous binding of two Mg^2+^ cations on both sides of a pentacoordinate intermediate stabilizes the transition state in catalysis, thereby facilitating the reaction [1]. However, the weakness of this hypothetical catalytic scheme is mainly derived from the crystal structure of alkali phosphatase, which requires Zn^2+^ rather than Mg^2+^ [2]. In fact, there is general agreement that Zn^2+^ possesses much stronger coordination properties with protein atoms than Mg^2+^. Despite such questions, this hypothesis, globally called the “two-metal-binding mechanism” has been accepted by many scientists in molecular biology, presumably because most Mg^2+^-dependent nucleases exhibit crucial functions in gene expression and regulation. In addition, this hypothesis has an attractive aspect in that it can reasonably explain the Mg^2+^-dependent polymerization reaction from the XTP substrate when assuming the putative reverse reactions. We presume that this implicit generality may have led to widespread acceptance of the “two-metal-binding mechanism” without definitive evidence [3,4].

In accordance with recent developments in cryo-electron microcopy (cryo-EM), many structural reports published to date for Mg^2+^-dependent nucleases or spliceosomes support the “two-metal-binding mechanism” as the hydrolytic scheme. However, the direct visualization of Mg^2+^, which is comparable to the electron density of H_2_O, requires high-resolution analyses to determine the exact Mg^2+^ positions on electron density maps, irrespective of whether X-ray crystallography or cryo-EM is used. For instance, a cryo-EM structural study of a spliceosome containing a major ribozyme was published several years ago [5]. In this report, the authors used Ca^2+^ as an inhibitor to halt cleavage, instead of catalytic Mg^2+^. Importantly, Ca^2+^ cations are well-known inhibitors of Mg^2+^-dependent protein nucleases as well as ribozymes. From the 3D structural viewpoints, immobile states are required to capture enzymatic actions. Therefore, we are inevitably obliged to introduce mutations within the enzyme active sites or use Ca^2+^ as an inhibitor, in order to visualize catalytic mechanisms. We assume that interpretations of the dynamic catalytic mechanism, based on the immobile states of original actions, are analogous to a philosophical antinomy. Thus, the purpose of this commentary is to critically reconsider the Mg^2+^-dependent catalytic reactions of nucleases and ribozymes, particularly focusing on our structural study on *Escherichia coli* (*E. coli*) ribonuclease HI (RNase HI).

### Background of RNase HI structural study

In 1990, a group led by one of the authors first reported the 1.8 Å X-ray crystal structure of RNase HI from *E. coli* [6]. The high-resolution structure revealed that the enzyme contained one active site, where one Mg^2+^ cation was bound to four conserved acidic residues, Asp10, Glu48, Asp70 and Asp134 (the DEDD motif), in addition to a notable basic protrusion. This structure is sufficiently unique to illustrate how a DNA/RNA hybrid duplex specifically binds to the enzyme. Heavier metals, such as Ca^2+^, Ba^2+^ and Co^2+^, soaked into crystals, were found to occupy the same site as Mg^2+^ [7]. As discussed later, the activator Mg^2+^ and inhibitor Ca^2+^ notably occupied the same site. On the other hand, Davies et al. published the 2.4 Å crystal structure of the RNase H domain of HIV-1 reverse transcriptase [8]. Although this domain showed no activity in vitro, the structure was overall similar to that of *E. coli* RNase HI, including the catalytic center of four conserved acidic residues (DEDD motif), and curiously, the soaked Mn^2+^ ions were bound to two different sites roughly 4 Å apart. This structural feature of metal binding contrasts with that of *E. coli* RNase HI. We considered that this discrepancy might be derived from artifacts in the soaking experiments; thus, our group attempted to co-crystallize RNase HI with Mg^2+^ at a concentration higher than 100 mM MgSO_4_. As expected, this structure clearly showed one Mg^2+^ cation within the acidic active site [9]. Our group has attempted to crystallize the complex of *E. coli* RNase HI with DNA/RNA hybrid duplexes, using various active-site mutants. However, all attempts were unsuccessful.

Nowotny et al. reported the crystal structure of human RNase HI complexed with an RNA/DNA hybrid in 2007 [10]. Human RNase HI is a protein derived from a mitochondrial gene [11]; thus, this enzyme exhibits high sequence identity (34%) with *E. coli* RNase HI, which allows a detailed 3D description of the *E. coli* RNase HI-RNA/DNA hybrid complex. Expectedly, human RNase HI contains a “basic protrusion” which plays major roles in binding the DNA strand of an RNA/DNA hybrid duplex [10]. In addition, the recognition mechanisms of nearly DNA A-type duplex substrates by the enzyme were revealed at the level of specific polar and hydrophobic interactions between amino acid residues and substrate nucleotides. Notably, a putative cleaved phosphate backbone was found to lie in the closest vicinity of Glu186 and Asn210 (Asp in the wild type), forming an active center for divalent metal binding, thereby reconfirming that these two acidic residues at least are involved in cleavage of the RNA backbone. We appreciate that this report provides a clear and complete view of the specific recognition between RNase HI and DNA/RNA hybrids. Consistent with the heteroduplex features of the substrate, the cleaved RNA strand faces the active site, whereas the DNA strand is bound to the specific binding channel of the basic protrusion. However, the descriptions of the enzymatic cleavage mechanism appear to be based on the classical two-metal-binding mechanism. The mechanism of Mg^2+^ action remains ambiguous, because the crystal structure contains Ca^2+^ as an inhibitor rather than Mg^2+^.

**Figure 1:**
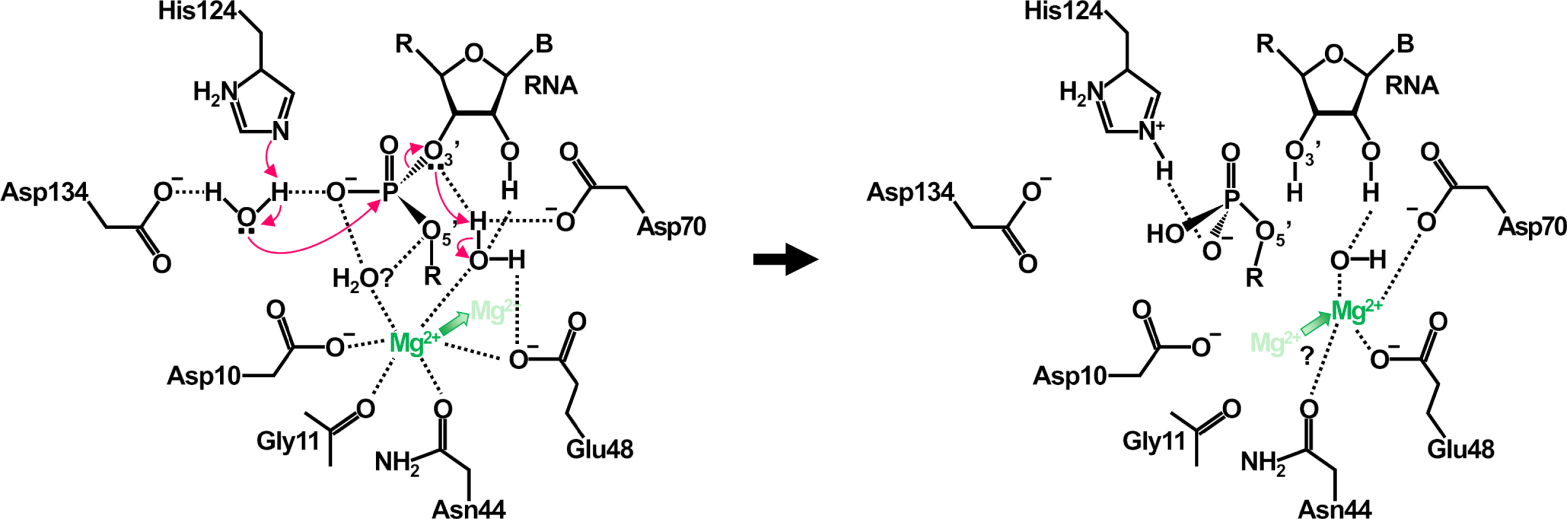
Hypothetical catalytic mechanism of *E. coli* RNase HI. This figure is revised from Fig. 6 in Kashiwagi et al. [6].

Since the first report of the *E. coli* RNase HI crystal structure [6], our group has mainly focused on solution studies of catalytic mechanisms, utilizing mutant proteins [12–15] and chemically modified nucleic acids [16–18] in combination with NMR. By summarizing these results and integrating them with high-resolution X-ray crystal structures of various protein mutants, our group proposed a new enzymatic scheme distinct from the classical two-metal-binding mechanism [19]. As shown in Fig. 1, RNase HI contains two metal-binding sites within one catalytic center consisting of the conserved DEDD motif and invariant His124. Importantly, His124 and Asp134 jointly activate the nucleophilic attack of water on the phosphate group. Simultaneously, proton donation prior to product release is mediated through a water molecule by the combined actions of Glu48, Asp70, Mg^2+^, and a 2'-OH group of the substrate ribose at the cleavage site. During this phosphoryl transfer reaction, a pentacoordinate intermediate at the phosphorus atom is formed, and the resulting increase in negative charge is compensated by the Mg^2+^ cation. This catalytic scheme assumes that only one metal ion operates, even though the enzyme contains two potential metal-binding sites: a single Mg^2+^ cation shifts between the two sites during the cleavage reaction. What is the driving force behind this shift of the metal ion? The binding of water molecules is likely stable at both sites as long as they remain in a neutral state. However, an energetically unfavorable situation may arise during the catalytic reaction, as the negative charge of generated hydroxide ion would be repulsive towards the Asp70 and Glu48 side chains. Thus, Mg^2+^ shifts from one side to the other to alleviate this repulsion. This interpretation of a single Mg^2+^ ion is supported by findings from kinetic studies using chemically modified substrates for hydrolysis [12,16,17,19].

## Retrospective comments on RNase HI study

### Generality of catalytic scheme in phosphoryl transfer reactions

The Mg^2+^-dependent phosphoryl transfer reaction can be divided into three steps: specific binding of polynucleotide substrates to the enzyme, backbone cleavage, and product release. As mentioned in the previous section, we proposed a catalytic mechanism in which the mobility of a single Mg^2+^ ion explains the catalytic mechanism of RNase HI [19]. However, since this initial proposal of the mobile metal ion hypothesis 30 years ago, numerous structural studies using X-ray crystallography and cryo-EM have supported the classical two-metal-binding mechanism for catalytic schemes in Mg^2+^-dependent nuclease-DNA or -RNA complexes without definitive evidence [3,4]. In this framework, the catalytic mechanism is explained as follows: two Mg^2+^ cations bind simultaneously on both sides of pentacoordinate intermediate, together with an additional anion, thereby facilitating cleavage of the polynucleotide backbone [1]. As described previously, atomic-level visualization of enzymatic processes requires frozen reaction states; therefore mutational protein targets or Ca^2+^ inhibitors must be used in structural studies. For instance, the correct identification of Ca^2+^ ions within spliceosomes using cryo-EM would be extremely challenging.

Furthermore, from the functional perspective of molecular biology, we must underscore an important issue: many structural studies use Mn^2+^ cations to identify of metal-binding sites on electron density maps, instead of Mg^2+^, whose electron density is comparable to water molecules. However, except under stress conditions, intracellular concentrations of free Mn^2+^ ions are so low that they are unlikely to have biological functions, therefore, the use of Mn^2+^ is considerable only as a probe for Mg^2+^ in structural analyses.

### Similarities between RNase HI and DNA restriction endonucleases

Restriction endonucleases are ubiquitous in prokaryotes. Their major biological function is to protect the host genome against specific bacteriophages by cleaving the backbone of foreign DNA duplexes. In particular, type II restriction endonucleases classified in the PD-(D/E)XK family require Mg^2+^ for cleavage of DNA backbones, whereas Ca^2+^ acts as a general inhibitor of DNA cleavage. These enzymatic properties are similar to those of RNase HI, apart from differences in the backbone of DNA or RNA. A German group led by Alfred Pingoud has conducted extensive biochemical studies on the catalytic mechanism of restriction endonucleases. They published a conclusive report, showing that Ca^2+^ mainly stabilizes binding of the pentacoordinate transition state but does not promote cleavage of the chemical bond, and the presence of two Ca^2+^ ions inhibits the reaction [20]. This report also concluded that type II restriction endonucleases have two metal-binding sites, thereby proposing a complex scheme termed the “activation/attenuation” mechanism. Notably, this term was first introduced in biochemical and structural studies of the cleavage mechanism of RNase HI [21]. This study suggested that RNase HI contains two Mn^2+^-binding sites, with the second site having an inhibitory role. More recently, our group reported higher-resolution crystal structures in association with Mg^2+^ and Zn^2+^ cations, one of which showed that Mg^2+^ cations bind to two distinct sites in the active center of *E. coli* RNase HI [22]. Comparison of the Mg^2+^- and Mn^2+^-bound structures showed that the two metals occupy identical positions at one site, whereas the second Mg^2+^ is slightly shifted along with an essential carboxyl side chain at the other site. Consequently, both metal cations are preserved within the same active-site architecture.

Thus, we propose that RNase HI and type II restriction endonucleases follow the same metal-dependent catalytic scheme. This interpretation is consistent with crystallographic studies of type II restriction endonucleases-DNA complexes [23]. Even in the catalytic mechanism of type II restriction endonucleases, a single Mg^2+^ cation is likely mobile between the two sites during catalysis, similarly to RNase HI.

### Structural study of the very short patch repair (Vsr) endonucleases

**Figure 2:**
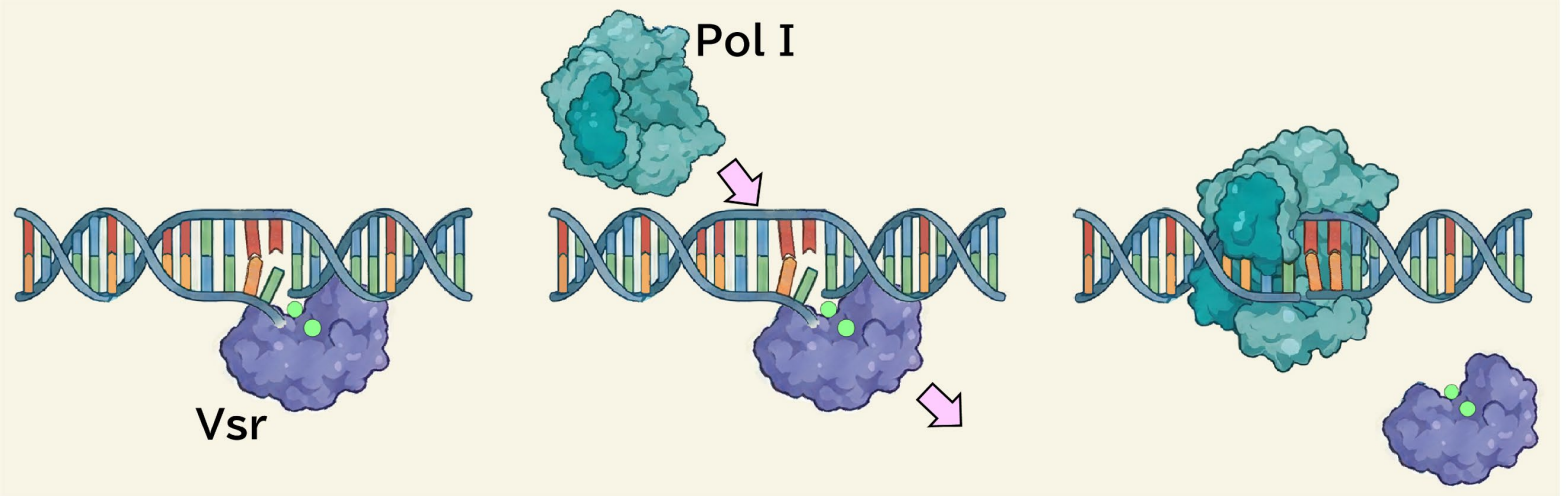
Hypothetical mechanism of exchange from Vsr to Pol I. Mg^2+^ ions are indicated by emerald green circles. The Mg^2+^-involved cluster determined from the crystal structure is shown in Fig. 5 in Tsutakawa et al. [24].

DNA repair involves plenty of Mg^2+^-dependent nucleases. Here, we describe the Vsr nuclease, which plays a major role in repairing TG mismatch base pairs in DNA duplexes. Specifically, the Vsr endonuclease recognizes a TG mismatch within a canonical Watson-Crick DNA duplex and cleaves at the 5' side of thymine, leaving a 5'-phosphate and a 3'-hydroxyl at the termini. This cleavage process is Mg^2+^-dependent; therefore, the Vsr endonuclease is classified as Mg^2+^-dependent nucleases. Our group reported a high-resolution crystal structure of Vsr complexed with Mg^2+^ and a DNA duplex containing a TG mismatch [24]. Notably, the catalytic center contains two Mg^2+^ cations, one of which is coordinated with the cleaved deoxyribose 3'-oxygen, suggesting that the DNA remains in an intermediate state and that the structure represents a state just before product release (see Fig. 5 in Tsutakawa et al. [24] for details). This observation raises the question of what drives product release. A clear answer was provided by a report, showing that DNA polymerase I (Pol I) releases Vsr endonuclease from the nicked DNA substrate [25]. Presumably, Pol I would be involved in an unknown function for dissociation of the Vsr endonuclease-nicked DNA complex (Fig. 2).

### Single mobile Mg^2+^-dependent apurinic/apyrimidinic endonuclease-1 (APE1)

**Figure 3:**
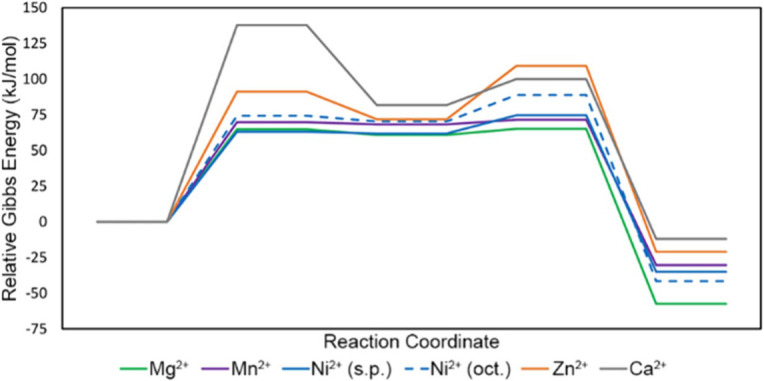
Relative Gibbs energy along the QM/MM predicted mechanism of action for APE1 activity in the presence of various metals. Reproduced with permission from Figure 5A in Ref. [27]. Copyright 2025 American Chemical Society.

Despite the widespread acceptance of the two-metal-binding mechanism, APE1 belongs to a group of nucleases whose hydrolysis depends on a single metal ion. This conclusion is based primarily on crystallographic studies combined with mutational experiments. We particularly highlight the one nuclease APE1, which plays crucial roles in DNA repair. Importantly, this nuclease appears to be a suitable target for molecular dynamics studies, such as large-scale quantum mechanics/molecular mechanics (QM/MM) modeling [26]. For instance, we cite an informative review in 2025 by Nikkel et al. [27]. In this review, a figure, showing the relative Gibbs energy plotted against the reaction coordinate is particularly intriguing (Fig. 3). This figure presents relative Gibbs energies calculated using QM/MM models for Mg^2+^, Mn^2+^, Ni^2+^, Zn^2+^, and Ca^2+^, respectively. Notably, the transition state with active Mg^2+^ is nearly flat, whereas that with inhibitory Ca^2+^ shows two barriers and an intermediate minimum. These features are consistent with experimentally observed activity of APE1.

In agreement with the QM/MM analysis described above, Oezguen et al. reported that molecular dynamics (MD) simulations of APE1 complexed with cleaved and uncleaved damaged DNA demonstrate movement of a single Mg^2+^ ion from one site to another [28]. Furthermore, these two sites correspond to the respective metal positions determined by X-ray analysis. Thus, they concluded that APE1 cleaves the substrate through a “moving metal mechanism”.

### Future perspectives

We conclude that RNase HI essentially requires a single Mg^2+^ cation to cleave the RNA strand of a DNA/RNA hybrid duplex, whereas Ca^2+^ strongly inhibits RNase HI. We sought to understand why these functional properties are conserved among many intranuclear nucleases and ribozymes, including spliceosomes. The distinct properties of these two metals are evolutionally conserved. In this context, it should be noted that the exchange rate of hydration water differs markedly between Mg^2+^ and Ca^2+^ (10^5^ s^‒1^ for Mg^2+^ and 10^8^–10^9^ s^‒1^ for Ca^2+^) [29]. We considered whether this difference in exchange rate could account for the inhibitory effect of Ca^2+^ on RNase HI cleavage. This question can be naturally extended to other nucleases and represents an important challenge for future MD studies.

Finally, we highlight general issues related to active-site conformational fluctuations during nuclease catalysis. The typical rate of phosphoryl transfer reactions is on the order of milliseconds; thus, time-resolved X-ray crystallography is not be well suited to capture these events because of limitations in experimental timescales and constraints imposed by the crystal lattice, although Samara and Yang reported RNase HI study using this approach [30]. According to transition state theory proposed by Jencks [31], phosphoryl transfer reactions of nucleases involve pentacoordinate intermediate states. Our attempts to co-crystallize the RNase HI-substrate complex in the presence of a stable intermediate mimic such as aluminum fluoride have been unsuccessful so far. Therefore, we wonder what methods allow direct observation of conformational fluctuations during the catalytic processes of Mg^2+^-dependent nucleases at relevant spatial scales.

## Conflicts of interest

The authors declare no competing financial interests.

## Author contributions

All authors wrote this manuscript.

## Data availability

The evidence data generated and/or analyzed during the current study are available from the corresponding author on reasonable request.
